# An association between anti-platelet drug use and reduced cancer prevalence in diabetic patients: results from the Vermont Diabetes Information System Study

**DOI:** 10.1186/1471-2407-10-289

**Published:** 2010-06-15

**Authors:** Chris E Holmes, Maria E Ramos-Nino, Benjamin Littenberg

**Affiliations:** 1 Hematology and Oncology, University of Vermont, Given Building Second Floor, Burlington, VT 05401, USA; 2 Department of Pathology, University of Vermont, Burlington, VT 05405, USA; 3 University of Vermont, 89 Beaumont Avenue, S459, Burlington, VT 05405, USA

## Abstract

**Background:**

Diabetes is associated with an increased risk of several malignancies. Both diabetic patients and patients with cancer have an increase in platelet reactivity and platelet activation has recently emerged as a potential mediator of cancer progression. Drug therapies, such as aspirin, that reduce platelet reactivity reduce both cardiovascular and cancer risk.

**Methods:**

We performed a cross-sectional analysis to assess the association between history of cancer and current anti-platelet drug use in a primary care population of adults with diabetes enrolled in the Vermont Diabetes Information System.

**Results:**

Self-reported characteristics, medical history, and a complete medication list were recorded on 1007 diabetic adults. Fifty percent of diabetic patients used an anti-platelet drug. In unadjusted analysis, no association was seen between anti-platelet drug use and cancer history (OR = 0.93; *P *= .70). Platelet inhibitor use was associated with a decreased patient-reported history of malignancy in a multivariate logistic regression adjusted for age, sex, body mass index, comorbidity, and number of medications (OR = 0.66; CI 0.44-0.99; *P *= .045). Similar odds of association were seen in both males and females, and for aspirin and non-aspirin platelet inhibitor therapy.

**Conclusions:**

Our data suggest an association between anti-platelet drug use and reduced cancer prevalence in patients with diabetes. Given the potentially large implications of our observations in the diabetic population, further studies are required to determine if this association is causal.

## Background

Epidemiologic studies have found an increase in cancer mortality and risk of several cancer types in persons with type 2 diabetes mellitus[1-4]. The association is likely multifactorial and may be mediated by insulin resistance and the metabolic syndrome as well as common life-style choices and obesity[5,6]. Patients with diabetes commonly display an increase in platelet reactivity [7], and large studies have suggested they derive particular cardiovascular benefit from platelet inhibition [8]. Accumulating evidence also suggests a role for platelet activation in cancer progression and an increase in platelet reactivity has been seen in patients with metastatic cancer [9-11].

The use of aspirin, an inhibitor of platelet reactivity, is associated with cancer prevention in two of the most common solid tumor malignancies affecting patients with diabetes: colorectal and breast cancer. The efficacy of aspirin in cancer prevention has been linked in part to inhibition of COX-2 (inducible) driven pathways of malignancy development (reviewed in [12]). In murine models, however, thrombocytopenia and platelet antagonists decrease tumor metastases indirectly supporting a plausible role for COX-1 platelet inhibition in cancer prevention and treatment [13,14].

Given the role of aspirin in cancer prevention in the general population, the increase in platelet reactivity in diabetics, and the emerging role of platelet activity in cancer progression, we explored the association between anti-platelet drug use and cancer history in persons with type 2 diabetes. We hypothesized that persons with type 2 diabetes taking anti-platelet drugs such as aspirin would have a decreased risk of cancer compared to diabetic persons not using aspirin.

## Methods

The Vermont Diabetes Information System was a randomized trial of a decision support system in diabetes [15]. All diabetic adults in 69 primary care practices in Vermont and adjacent New York were enrolled. Following ascertainment of informed consent, a randomly selected subset of 1007 subjects completed a survey and a home visit by trained researchers between July 2003 and March 2005. Patients were asked to gather all current mediations, including over the counter drugs. Staff recorded the drug name, dose, frequency, and route of administration from each container. Duration of therapy was not ascertained. Weight and height were recorded using a portable stadiometer and scale.

Patient characteristics were obtained by questionnaire. Comorbidity (in addition to diabetes) was determined using a modification of the Self-Administered Comorbidity Questionnaire [16]. Patients who reported a history of any malignancy, including leukemia or lymphoma, were classified as having cancer. Dates of diagnosis, treatments, and site were not recorded. A1C was measured using high performance liquid chromatography or a Bayer DCA 2000 immunoassay point of care instrument (less than 1% of tests). Anti-platelet drug use was defined as daily use of aspirin (at least 75 mg), clopidogrel, ticlopidine, or cilostazol or a combination of these medications. The specific indication for anti-platelet therapy was not recorded.

We performed a cross-sectional association analysis of the interviewed subjects at the time of their enrollment in the VDIS trial. We explored the association between cancer and the use of anti-platelet agents, using logistic regression with history of cancer as the outcome variable and the use of anti-platelet drugs as the primary predictor variable. We then adjusted for possible confounding by social and clinical factors. Potential confounders tested were: gender, age, race (white/other), glycosylated hemoglobin level (A1C;mg%), insulin use (yes/no), body mass index (BMI;kg/m^2^), alcohol drinking (yes/no), cigarette smoking (yes/no), income (in eight ordered categories), duration of diabetes in years, high comorbidity (two or more comorbid conditions, number of medications, physical function status and mental function status. To reduce the number of variables in the final model, we excluded potential confounders that were associated with the outcome in univariate analysis with P > 0.2. The final multivariate analysis adjusted for age, sex, body mass index, presence of multiple co-morbidities and number of medications. In secondary analyses, we studied selected subgroups of patients based on sex and use of different platelet inhibitors. Association was expressed as the odds ratio (OR) with 95% confidence interval (CI).

All research was carried out according to the Helsinki declaration per the University of Vermont Institutional Review Board.

## Results

Selected characteristics of the study population (1,007 subjects) based on anti-platelet drug users versus non-users are described in Table 1. Half of the population were seniors, two thirds were obese, and 48% had an A1C > 7%. There was little racial diversity, reflecting the local population. Thirteen percent of patients reported a history of malignancy. Half of the patients used a platelet inhibitor (aspirin or non-aspirin platelet inhibitor). Aspirin use in this population has previously been found to be more prevalent in subjects with coronary artery disease and in men [17].

**Table 1:** Characteristics of 1007 study subjects

	*n*	*Mean*	*Range*	*n*	*Mean*	*Range*	*P*
**Anti-platelet therapy**	**Absent**			**Present**			
Age in years	499	62.1	22.2-89.5	508	67.6	36.0-92.6	< .001
Gender: male	499	39.7%		508	51.6%		< .001
Race: white,	497	96.6%		507	98.0%		.15
Body mass index in kg/m^2^	492	34.5	16.1-64.6	502	33.1	15.8-68.0	.003
Glycosylated hemoglobin A1C	497	7.1%	4.0-13.5	506	7.1%	4.9-12.9	.85
More than 1 comorbid condition	499	45.1%		508	53.5%		.007
Current smoker	498	18.5%		508	15.4%		.19
Number of medications	499	7.7	0-29	508	9.8	1-29	< .001
Self-reported history of malignancy	499	13.0%		508	12.2%		.70

**History of cancer**	**Absent**			**Present**			
Age in years	880	65.2	22.2-91.7	127	69.1	34.5-92.6	< .001
Gender: male	880	46.2%		127	41.7%		.34
Race: white,	878	97.3%		126	97.6%		.82
Body mass index in kg/m^2^	868	34.0	15.8-68.0	126	32.6	19.7-53.8	.05
Glycosylated hemoglobin A1C	876	7.1%	4.0-13.5	127	7.0%	4.8-13.3	.13
More than 1 comorbid condition	880	46.2%		127	71.7		< .001
Current smoker	879	17.8%		127	11.0%		.06
Number of medications	880	8.6	0-29	127	9.9	0-29	.003
Anti-platelet therapy	880	50.7%		127	48.8%		.70

In unadjusted analysis, no significant association was seen between anti-platelet drug use and cancer history (OR = 0.93; CI = (0.64, 1.34); *P *= .70), Table 1. However, an unadjusted analysis revealed a significant association between cancer history and age, more than one comorbid condition, BMI and number of prescriptions medications (p ≤ 0.05). In this same analysis, sex, ethnicity, use of insulin therapy, hemoglobin A1C, alcohol use, median income, duration of diabetes, number of anti-diabetic medications and physical functional status were not associated with a diagnosis of cancer.

A multivariate analysis adjusting for confounding variables revealed significantly lower odds of a cancer history among anti-platelet drug users (OR = 0.66; CI 0.44-0.99, Table 2). Because aspirin use was more common in men and some cancers are sex-associated, we repeated the analyses for each sex. The magnitude of the association was maintained in both males and females; however, the confidence intervals broadened with the smaller sample size. The association between anti-platelet drug use and reduced cancer history was not maintained in persons under the age of 65 years but was of significance in persons over the age of 65 years (P = 0.047).

**Table 2:** Association between platelet inhibitor therapy and history of cancer

*Population*	Multivariate (Adjusted) Analyses*			
	
	*N*	*OR*	*(95% CI)*	*P*
All subjects	994	0.66	(0.44, 0.99)	.045
Women	540	0.63	(0.36,1.08)	.09
Men	454	0.71	(0.38,1.32)	.29
Age < 65 years	477	0.93	(0.46, 1.90)	.85
Age 65 years or more	530	0.61	(0.37, 0.99)	.047
Excluding users of non-aspirin inhibitors	925	0.67	(0.44, 1.03)	.07
Excluding users of aspirin	525	0.64	(0.22, 1.80)	.39

*Adjusted for age, sex, body mass index, presence of ≥ 2 co-morbidities, and number of medications. Body mass index was not available for 13 subjects who were excluded from the multivariate analyses.

The individual anti-platelet drugs studied have distinct characteristics. For instance, aspirin inhibits platelets and inhibits COX pathways in tissues and tumors but the others do not. To exclude the possibility that the apparent protective effect of these medications is due to a medication-specific characteristic (other than anti-platelet activity), we divided the treatments into two groups (aspirin and non-aspirin platelet inhibitors) and repeated the analysis excluding any subjects who used one or the other of the groups (Table 2). Although the smaller sample sizes again reduced precision, the odds ratios changed little, indicating that the association is not due to some specific characteristic of one group of platelet inhibitor.

## Discussion

Platelet inhibitors were significantly associated with reduced odds of a cancer history in adults with diabetes in our study population. The association was not due to confounding by age, sex, obesity, or number of medications or co-morbidities. The odds of association were similar in men and women and for both aspirin and non-aspirin platelet inhibitor users although smaller numbers broadened the observed confidence intervals. Our original hypothesis was based on data from multiple studies supporting the use of aspirin therapy in cancer prevention in selected populations. We chose to focus on patients with diabetes because they have an increased reported cancer risk, an increase in platelet reactivity and a clear benefit from anti-platelet therapy in the cardiovascular setting. Consistent with our hypothesis, we found a reduction in reported cancer history was associated with anti-platelet drug use in a community based diabetic population.

The strong association between platelet inhibitors and a history of cancer is apparent only when controlling for other factors such as age, sex, obesity, and burden of comorbid conditions. This demonstrates that one or more of these factors confounds the relationship between platelet inhibition and cancer. As in all non-experimental studies, the possibility of additional, unmeasured, confounders that further influence the relationship between platelet inhibition and cancer cannot be excluded. Additionally, our population was heterogeneous with regards to cancer type and larger studies and/or cancer specific studies will be needed to determine an association (if any) between specific cancer subtypes and aspirin use in patients with diabetes.

These results are consistent with prior studies in selected patient populations. For example, aspirin use prevents the development of colorectal adenomas in patients with a prior history of these lesions [18]. In addition, several studies have suggested that aspirin use is associated with a reduced risk of breast cancer[19,20]. However, not all studies of aspirin have demonstrated a benefit in cancer prevention [21]. Our results are the first to focus on a diabetic population.

We found no difference in our study between male and female users of platelet inhibitor therapy. This is consistent with the finding that men and women have similar reductions in platelet reactivity to low dose aspirin therapy [22]. In addition, reductions in cancer risk associated with aspirin therapy are seen in gender specific cancers such as prostate cancer and breast cancer [20,23].

Mechanisms that explain the possible association between anti-platelet therapy and cancer were not addressed by this study. Although aspirin has COX- and prostaglandin-mediated tissue effects, the other agents studied do not, suggesting the effects might be related to platelet inhibition. In the laboratory, ticlopidine (one of two non-aspirin platelet inhibits used in our patient population) has been reported to inhibit pulmonary metastasis of melanoma as well as metastasis in a Lewis lung carcinoma model [24]. No human studies using these agents to prevent or treat cancer have been reported. Interestingly, some inhibitors of platelet function such as glycoprotein IIB-IIIA inhibitors have anti-tumor activity in animal models and have been found to have greater platelet inhibitor activity in patients with diabetes than non-diabetic patients [25].

This study provides the first evidence to suggest anti-platelet therapy may have an anti-cancer benefit in patients with diabetes. However, the conclusions are limited by the cross-sectional design, racial homogeneity of the subjects, use of unconfirmed cancer diagnoses, and lack of data on cancer site. In addition, anti-platelet drug duration or dose of therapy was not available and the temporal relationship between medication use and cancer diagnosis is unknown. While the majority of patients in our study are anticipated to have Type II diabetes, we did not collect this data directly and; therefore, were unable to determine if the demonstrated association is the same for both type I and type II diabetics.

## Conclusions

We found anti-platelet drug use was associated with a reduced number of reported cancer diagnoses in adults with diabetes who participated in the Vermont Diabetes Information System Study. Our results suggest targeting patient populations with increased platelet activation, such as patients with diabetes mellitus, may be a useful strategy to move platelet inhibitors successfully into the cancer prevention or treatment setting. Our results will need confirmation in larger, prospective studies given the cross-sectional design of the study. If confirmed, our results suggest patients with diabetes may receive a dual health benefit from a single, inexpensive drug.

## Abbreviations

COX: cyclo-oxygenase; VDIS: Vermont Diabetes Information System; A1C: glycosylated hemoglobin level; CI: confidence interval; OR: odds ration

## Competing interests

The authors, CH and MR, declare that they have no competing interests. BL is an officer and shareholder of Vermedx, Inc., which distributes the Vermont Diabetes Information System.

## Authors' contributions

CH conceived the study hypothesis, participated in data and study analysis and interpretation; MR aided in data analysis; BL established the VDIS study, performed data analysis and participated in study analysis and interpretation. All authors read and approved the final manuscript.

## Pre-publication history

The pre-publication history for this paper can be accessed here:

http://www.biomedcentral.com/1471-2407/10/289/prepub
